# The Homophilic Domain – An Immunological Archetype

**DOI:** 10.3389/fimmu.2016.00106

**Published:** 2016-03-21

**Authors:** Heinz Kohler, Jagadeesh Bayry, Srinivas V. Kaveri

**Affiliations:** ^1^Department of Microbiology Immunology, University of Kentucky, Lexington, KY, USA; ^2^Institut National de la Santé et de la Recherche Médicale Unité 1138, Paris, France; ^3^Centre de Recherche des Cordeliers, Equipe – Immunopathologie et immuno-intervention thérapeutique, Paris, France; ^4^Sorbonne Universités, UPMC Univ Paris 06, UMR S 1138, Paris, France; ^5^Université Paris Descartes, Sorbonne Paris Cité, UMR S 1138, Paris, France

**Keywords:** natural antibodies, homophilic, immunoglobulins, idiotype, induced antibodies, T15, 1F7

## Abstract

The homophilic potential emerges as an important biological principle to boost the potency of immunoglobulins. Since homophilic antibodies in human and mouse sera exist prior environmental exposure, they are part of the natural antibody repertoire. Nevertheless, hemophilic properties are also identified in induced antibody repertoire. The use of homophilicity of antibodies in the adaptive immunity signifies an archetypic antibody structure. The unique feature of homophilicity in the antibody repertoire also highlights an important mechanism to boost the antibody potency to protect against infection and atherosclerosis as well to treat cancer patients.

## Natural Antibodies – The Virgin Immune Response

Natural antibodies are immunoglobulins in sera of mammalians that are neither immunologically challenged nor responding. For example, natural antibodies are in sera of neonatal or germ-free mice. Natural antibodies are of IgM, IgG, and IgA isotypes and play an important role in the immune homeostasis and protection against pathogens (1–6). B1 cells in mice produce natural antibodies (7, 8). In human, CD20^+^CD27^+^CD43^+^ memory B cells were recently identified as murine B1 counterpart (9). Natural antibodies are the important components of the therapeutic intravenous immunoglobulin (IVIG) (10–12) that is widely used in the therapy of autoimmune and inflammatory diseases (13–17). These functions of natural antibodies also indicate diverse roles played by B cells in the immune homeostasis (18–22).

## The Idiotypic Interactions

Oudin and Jerne are the founders of the idiotype concept (23, 24). They “discovered” that one antibody could recognize another antibody as an individual and unique member of the immune system. The uniqueness of an antibody lies in the variable region of heavy and light chains and has been named idiotype. The idiotype antibody individuality is separated from the antigen-binding site, also determined by unique sequence variability. However, both regions are linked by a unique variability and have a functional relation. Identifying an antigen-binding site also reveals the linked idiotype and *vice versa*, an idiotope reveals the associated antigen specificity. However, there are important exceptions to this linkage rule: the so-called common idiotopes (IdX) are expressed on antibodies with different antigen specificities (25). Furthermore, antigen-binding site and idiotype site can be identical or overlapping.

Studies on the functional interactions of anti-idiotype binding to antibodies have established a classification in the idiotype field. Jerne termed anti-idiotypes that do not interfere with the antigen-binding site as Ab2α and anti-idiotypes that block the antigen binding as Ab2β (26). Ab2 was further differentiated by adding the term Ab2γ to describe Ab2 that only partially inhibits antigen binding (27, 28). The Ab2β has another unique functional feature. It mimics the antigen by its perfect fit into another antigen-binding site. Ab2β has been used as an antigen to induce a specific immune response (28). Another shared idiotype has been discovered in HIV-infected primates (29). This anti-idiotype antibody has been termed as Ab2δ (30).

In mice, “prime” antibodies lack the dominant expression of certain shared idiotype, such as the so-called T15 idiotype, that develops weeks after birth without environmental challenge (31, 32). Therefore, the T15 dominance can be considered a part of the natural antibody repertoire. The T15 idiotype is also present in primates, including man (33). Perhaps a review of the idiotypic circuits is helpful for the understanding of the biological and immunological properties of the T15 archetypic idiotype. A hallmark of the T15 antibody family is their ability to self bind for producing homophilic complexes (34–36). The domain responsible for antibody self-binding (homophilicity) has been described as a region in VH of T15, extending from CDR2 to Fr3 (35, 37). Because of their preimmune presence in normal sera, homophilic antibodies are segment of the natural antibody repertoire (33, 37).

## The Biology and Immunoregulatory Functions of Homophilic Antibodies

The homophilic domain in antibodies was identified, and peptides that can confer the homophilic effect to other antibodies were made (38). Further studies showed that the homophilic domain can be expressed independently from the specificity of the antigen-binding site (39). This suggested that any antibody could be made homophilic by attaching the homophilic domain. In early experiments, a chemical affinity conjugation method was introduced to make the homophilic Ig (40); later, recombinant techniques were used to produce a homophilic antibody fusion protein.

Human and mouse sera contain antibodies that express the homophilic domain (33), confirming that homophilic antibodies are part of the so-called natural antibodies (41). Homophilic antibodies with specificity for phosphorylcholine (PC) are superior in protecting against *Streptococcus pneumoniae* infection (42). Homophilic anti-PC antibodies are also highly effective to reduce atherosclerotic plaque formation (43, 44) and perhaps have other housekeeping functions. Antigenicity of the homophilic domain was tested in mice, rabbits, and non-human primates by injecting the homophilic domain peptide and treating macaques with a homophilic anti-CD20 antibody (45). No antibodies could be detected against the homophilic domain. This finding suggests that homophilic antibodies could be used as therapeutic drugs to treat human diseases without allergic side effects (46).

Indeed, human natural antibodies with specificity for PC protect against atherosclerotic plaque formation by binding to oxidized LDL and inhibit macrophage activation (43, 47). Immunizing atherosclerotic mice with PC antigen reduces plaque formation (44). Silverman and colleagues showed that SLE patients with low natural antibodies develop more serious disease (48). Emerging evidence suggested that natural antibodies could control transplant rejection (49).

Another “natural” antibody in normal human sera has been described with specificity for HLA (50). This anti-HLA is not detectable in sera but is revealed after IgG purification. The homophilic T15 peptide from the complementarity-determining region/framework masked the HLA recognition of non-purified IgG. Since the T15 homophilic peptide dissociate the homophilic Ig complex, thereby reducing the polyvalency and binding potency, one can conclude that in sera, auto-anti-HLA antibodies are bound to corresponding HLA and are not detected. It follows that natural auto-anti-HLA antibodies are homophilic. Thus, the homophilic property of antibodies plays a role in the functional regulation of antibodies either by masking them or by exposing them under appropriate conditions.

A homophilic state of the Ig B-cell receptor (BCR) has been described in chronic lymphocytic leukemia (51, 52). Peptides from the VH Ig BCR bind to the BCR providing the basis for self-recognition of the BCR. Recently, we used the T15 homophilic peptide to inhibit proliferation of murine human B-cell lines (46).

## The Unique Biophysical Properties of Homophilic Antibodies

Working with homophilic-converted Trastuzumab (Herceptin), we discovered that the dose–response in inducing apoptosis in a Her2/neu-expressing human cell line was not linear but was bell shaped (53). The highest concentration tested did not induce the highest amount of apoptosis, but a lower concentration produced the most apoptosis. Similar non-linear dose effects were observed in fluorescence staining of tumor cells. This paradoxical dose effect was also observed in xenograft experiment (54).

This non-linear dose affecting the potency of homophilic Herceptin could be due to the inherent mechanic of self-binding that was observed in 1986 (37, 55). Here, self-binding of the T15 antibody decreases as the concentration of homophilic antibody increases, producing a bell-shaped pattern. This indicates that the equilibrium of self-bound antibody and free antibody is controlled by the concentration of homophilic antibody. Thus, the lattice of build up of homophilic antibodies at the tumor target cell would decrease at a higher concentration, thereby reducing apoptosis. This paradoxical effect has been observed with homophilic anti-Her2/neu (Herceptin) (54). In this study, we showed that the viscosity of homophilic antibodies increases with temperature and decreases at lower temperature, an effect not observed with the most other proteins or organic compounds. Furthermore, the binding to antigen by a homophilic antibody is also higher at physiological temperature than by the non-homophilic parental antibody. Lastly, the binding of homophilic Herceptin to tumor cells is concentration dependent, whereby a lower concentration targets better than a higher concentration. The unique biophysical properties of homophilic antibodies represent another aspect in the natural antibody repertoire.

## Homophilicity in Shared Idiotypes

We believe that we are only at the beginning to discover networks of shared idiotypes. By using polyclonal anti-idiotype antibodies, Urbain and colleagues have observed shared idiotypes expressed on antibodies with different antigen specificity in rabbits and mice (26, 56). The monoclonal 1F7 anti-idiotype recognizes antibodies against viral antigens and is first described in humans (29, 57, 58). The T15 and 1F7 idiotypic networks harbor important biological and medical properties: dominant T15 antibodies protect against pneumococcal infection (59) and perform a housekeeping function to reduce atherosclerotic plaque formation (43); the 1F7 idiotope is expressed on antibodies against HIV-1 (57, 60) and hepatitis C (61, 62) (see also contribution by Muller et al.). Furthermore, 1F7-positive human anti-HIV-1 antibodies carry the homophilic domain (Veljkovic and Kohler, unpublished). These studies on homophilic antibodies show that homophilicity is utilized in the innate immunity (natural antibodies) and by the adaptive immune response.

## Homophilicity as a General Concept in System Biology of Natural Antibodies

The data on homophilic natural antibodies point to a general strategy of enhancing potency not only of antibodies but also other biological systems (63). This finding can be viewed as a paradigm in System Biology in the T15 and 1F7 idiotypic networks. Both systems utilize the antibody homophilicity. Both systems also use this property to achieve a high level of antibody potency. Antibodies with T15 expression in normal sera are close to germline encoding: (i) the T15 dominance develops late in ontogeny (31), (ii) the homophilic domain contains H2 mutations, (iii) analysis of the neonatal repertoire by Coutinho (64) shows only evidence for extensive idiotypic network and for homophilic binding, and (iv) homophilic antibodies have been detected after stimulation (38, 46). Furthermore, in support of the adaptive creation of the homophilic domain, the sequence of homophilic domains is found different (51, 52). Thus, certain homophilic antibodies are not part of the so-called natural antibodies but are induced by antigenic stimulation.

The homophilic antibody enhancement and therapeutic potential have been already demonstrated (38, 46). The native dominant expression of 1F7 idiotype on anti-HIV-1 antibodies may lead to a novel vaccine concept (65). There is one important difference between the T15 and the 1F7 system: T15 antibodies are present in preimmune serum without external antigen exposure; 1F7 antibodies recognizing anti-HIV and anti-HBC antibodies are induced after infection and are not natural antibodies. Nemazee et al. recently promoted the idea of targeting germline antibodies for inducing a maturation sequence, leading to broadly neutralizing Abs against HIV-1 (66). The use of a germline targeting immunogen is different from the concept of stimulating B-cells with idiotype expressing receptors. Prior to triggering, these B-cells do not produce detectable antibodies and do not contribute to the natural antibody repertoire (57). Using the 1F7 antibody as vaccine would not require additional immunogen(s) to trigger the antibody maturations process, since the 1F7 idiotype is expressed by potent broadly neutralizing Abs (Parsons, unpublished). HIV-1 infection is expected to stimulate the expanded clones of 1F7 expressing B-cells to produce broadly neutralizing Abs (57).

## Summary

The discussion of the unique feature of homophilicity as a part of the natural and induced antibody repertoires (65) highlights an important mechanism to boost the antibody potency to protect against infection and atherosclerosis (44) as well to treat cancer patients (46). The concept of homophilicity is introduced here to describe the unique coexistence and synergism of acquired immunity with innate immunity. The homophilicity of antibodies in the natural and acquired repertoires emerges as an archetypic principle in the immune system.

## Author Contributions

HK surveyed the literature and wrote the article. JB and SK provided suggestions. All authors approved the final version of this article for publication and accepted the responsibility for the integrity of the work.

## Conflict of Interest Statement

The authors declare that the research was conducted in the absence of any commercial or financial relationships that could be construed as a potential conflict of interest.
